# MUC4-promoted neural invasion is mediated by the axon guidance factor netrin-1 in PDAC

**DOI:** 10.18632/oncotarget.5668

**Published:** 2015-09-15

**Authors:** Linjun Wang, Xiaofei Zhi, Yi Zhu, Qun Zhang, Weizhi Wang, Zheng Li, Jie Tang, Jiwei Wang, Song Wei, Bowen Li, Jianping Zhou, Jianguo Jiang, Li Yang, Hao Xu, Zekuan Xu

**Affiliations:** ^1^ Department of General Surgery, The First Affiliated Hospital of Nanjing Medical University, Nanjing, Jiangsu, P.R. China; ^2^ Department of General Surgery, The Affiliated Hospital of Nantong University, Nantong, Jiangsu, P.R. China; ^3^ Department of Gastrointestinal Surgery, The Affiliated Yixing Hospital of Jiangsu University, Yixing, Jiangsu, P.R. China; ^4^ Department of Gastrointestinal Surgery, Taizhou People's Hospital, Taizhou, Jiangsu, P.R. China; ^5^ Collaborative Innovation Center for Cancer Personalized Medicine, Nanjing Medical University, Nanjing, Jiangsu, P.R. China

**Keywords:** MUC4, neural invasion, pancreatic ductal adenocarcinoma, netrin-1

## Abstract

Neuralinvasion (NI) is an important oncological feature of pancreatic ductal adenocarcinoma (PDAC). However, the underlying mechanism of NI in PDAC remains unclear. In this study, we found that MUC4 was overexpressed in PDAC tissues and high expression of MUC4 indicated a higher NI incidencethan low expression. *In vitro*, MUC4 knockdown inhibited the migration and invasion of PDAC cells and impaired the migration of PDAC cells along nerve in dorsal root ganglia (DRG)-PDAC cell co-culture assay. *In vivo*, MUC4 knockdown suppressed the NI of PDAC cells in a murine NI model. Mechanistically, our data revealed that MUC4 silencing resulted in decreased netrin-1 expression and re-expression of netrin-1 in MUC4-silenced cells rescued the capability of NI. Furthermore, we identified that decreased netrin-1 expression was owed to the downregulation of HER2/AKT/NF-κB pathway in MUC4-silenced cells. Additionally, MUC4 knockdown also resulted in the downregulation of pFAK, pSrc, pJNK and MMP9. Taken together, our findings revealed a novelrole of MUC4 in potentiating NI via netrin-1 through the HER2/AKT/NF-κBpathway in PDAC.

## INTRODUCTION

Pancreatic cancer (PC) is the fourth leading cause of cancer-related deaths worldwide [1]. The median survival of PC patients is approximately 4.1 months, with a 5-year survival rate lower than 5% [2]. The poor PC outcomes are on account of the lack of early diagnostic markers and effective treatment [3]. Even after curative resection, the recurrence rate is as high as 93.6% on account of metastasis [4]. Thus, it is important to identify novel therapeutic targets for better treatment of PC.

Neural invasion (NI) is extremely common in PDAC and is observed in approximately 71% to 98% of PC specimens [5, 6]. Previous studies have shown that NI is an important prognostic factor for PDAC [7]. In addition, NI is strongly associated with local recurrence after curative tumor resection and is a major cause of neuropathic pain [8]. However, few studies have investigated the molecular mechanisms underlying NI in PDAC.

MUC4 is a membrane-anchored glycoprotein originally discovered in a highly metastatic rat mammary adenocarcinoma [9]. Since then, MUC4 has been found aberrantly expressed in multiple human cancers, including lung, breast, gall bladder, prostate, ovarian, biliary tract and pancreas [10-15]. Our team previously demonstrated that MUC4 induced the nuclear translocation of β-catenin and potentiated cell growth, metastasis and angiogenesis in pancreatic cancer [16]. In addition, we also showed that MUC4 induced the apoptosis of antigen-specific cytotoxic T lymphocytes and promoted tumor immune escape in pancreatic cancer [17]. In this study, we identified a new role of MUC4 in NI and revealed the underlying mechanisms.

## RESULTS

### MUC4 is overexpressed in PDAC tissues, and its expression correlates with NI

**Figure 1:**
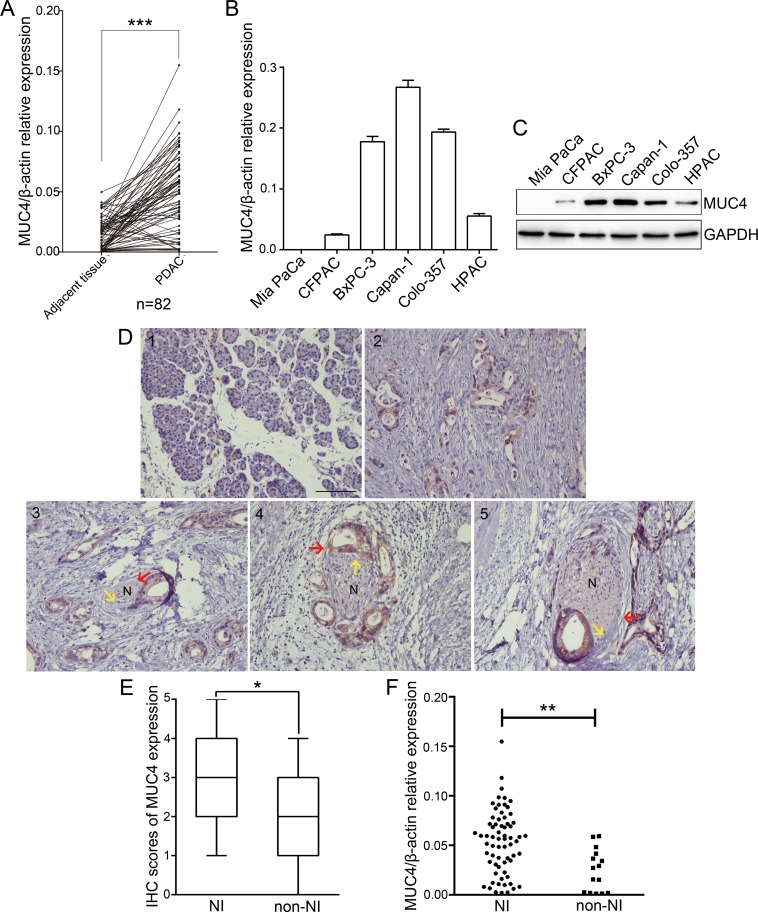
MUC4 expression is increased in PDAC and correlates with NI **A.** Real-time RT-PCR analysis of MUC4 mRNA levels in 82 PDAC tissues and paired adjacent tissues. **B.** Real-time RT-PCR analysis of MUC4 mRNA levels in PDAC cell lines. **C.** Western blot analysis of MUC4 protein levels in PDAC cell lines. **D.** Immunohistochemical staining of MUC4 in normal pancreatic and PDAC tissues. (1) Negative staining in normal pancreatic tissue; (2) positivestaining in PDAC tissues and localization at the cell membrane; (3, 4, 5) three pathological stages of NI in PDAC tissues: (3) epineural tumor associations (ENAs) (i.e., lesions in which the cancer cells are directly touching the epineural sheet without penetrating the perineurium); (4) perineural invasion (PNI) (i.e., infiltration of cancer cells within the perineurium); (5) endoneural invasion (ENI) (i.e., infiltration of cancer cells into the endoneurium). N indicates nerve, red arrow points to the perineurium of the nerve, yellow arrow points to the endoneurium of the nerve, and brown staining at the cell membrane indicates PDAC cells. Original magnification, 200×; scale bar =100 μm. **E.** Box plot showing the immunohistochemistry (IHC) scores for MUC4 protein expression in 67 PDACtissues with NI and in 15 PDAC tissues without NI (non-NI). Differenceswere analysed using the Mann-Whitney *U*-test. **F.** MUC4 mRNA expression in 67 PDAC tissues with NI and in 15 PDAC tissues without NI (non-NI). The data are presented as mean ± SEM. *p < 0.05, **p < 0.01, ***p < 0.001.

We determined MUC4 expression in 82 human PDAC tissue samples and their matched adjacent tissues by real-time PCR. Our data showed that MUC4 mRNA levels in PDAC tissues were significantly higher than those in the adjacent tissues (Figure 1A). In addition, we also assessed the levels of MUC4 mRNA and protein expression in PDAC cell lines. As observed in Figure 1B and 1C, Capan-1 cell line expressed MUC4 at high levels, Colo-357 and BxPC-3 cell lines expressed MUC4 at moderate levels, HPAC and CFPAC cell lines expressed MUC4 at low level, while no expression was observed in Mia PaCa cell line. Next, we determined MUC4 protein expression levels in human tissues by immunohistochemistry and found that MUC4 protein expression was almost absent in normal pancreatic tissue but elevated inPDAC tissues (Figure 1D, Supplementary Figure S1). Moreover, we found that MUC4 protein expression was higher in PDAC tissues displaying NI compared to non-neurally invasive PDAC tissues (Figure 1E). The analysis of MUC4 mRNA expression levels also supported this result (Figure 1F). The correlation between MUC4 expression and clinicopathological characteristics was further analysed. PDAC tissues with more than two-fold enrichment in MUC4 mRNA levels (relative to adjacent tissues) were defined as the high expression group, whereas those with a less than two-fold increase or with a slight decrease in MUC4 mRNA levels were defined as the low expression group. As shown in Table 1, MUC4 expression significantly correlated with the T grade, stage and NI (p = 0.008, p = 0.027 and p = 0.001, respectively). NI was observed in 90.2% (55/61) of patients with high levels of MUC4 expression and in 57.1% (12/21) of patients with low levels of MUC4, suggesting a correlation between high MUC4 expression and high NI incidence. Collectively, our data showed that MUC4 is overexpressed in PDAC tissuesand that its expression correlates with NI.

**Table 1:** Correlation between clinicopathological factors and MUC4 mRNA expression in PDAC patients

Factor	Number (%)	MUC4 expression		*p*-value
		Low group	High group	
Age(years)				
≥60	50 (61.0)	12	38	0.676
<60	32 (39.0)	9	23	
Gender				
Male	48 (58.5)	11	37	0.507
Female	34 (41.5)	10	24	
Histological grade				
Well differentiated	10 (12.2)	2	8	0.378
Moderately differentiated	63 (76.8)	15	48	
Poorly differentiated	9 (11.0)	4	5	
T grade				
T1+T2	31 (37.8)	13	18	0.008**
T3+T4	51 (62.2)	8	43	
Lymph node metastasis				
Negative	35 (42.7)	12	23	0.120
Positive	47 (57.3)	9	38	
Stage				
I/II	34 (41.5)	13	21	0.027*
III/IV	48 (58.5)	8	40	
Serum CA19-9 (kU/L)				
≤ 39	20 (24.4)	3	17	
> 39	62 (75.6)	16	46	0.379
Neural invasion				
Absent	15 (18.3)	9	6	0.001**
Present	67 (81.7)	12	55	

Correlations were estimated by the Fisher's exact test,

* p < 0.05

** p < 0.01.

### MUC4 knockdown inhibits the migration and invasion of PDAC cells *in vitro*

**Figure 2:**
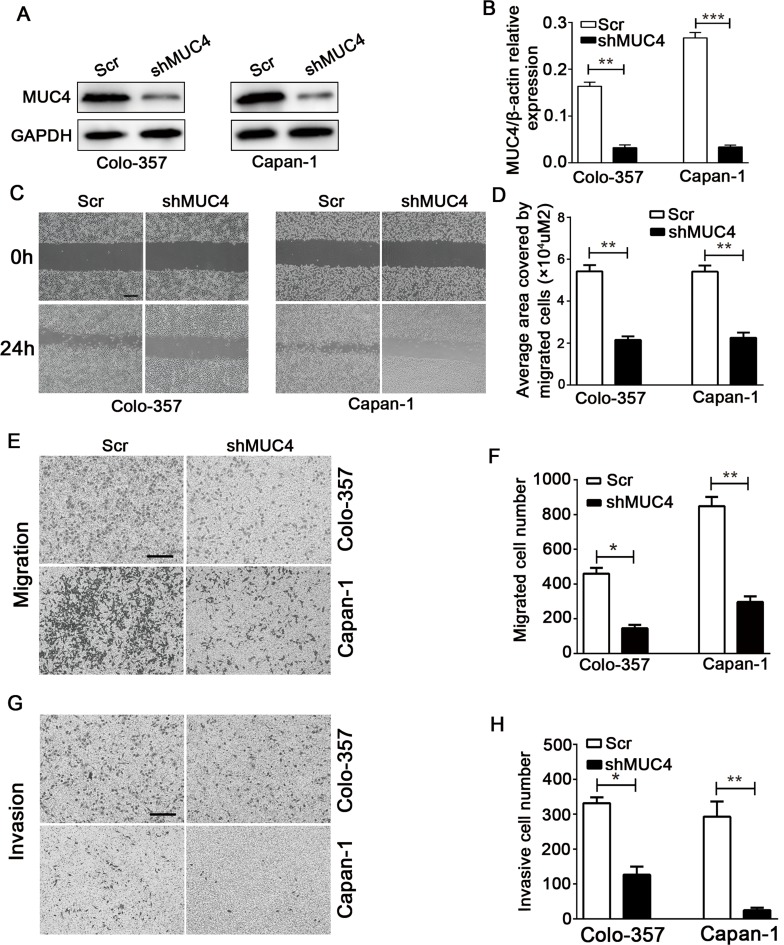
MUC4 knockdown inhibits the migration and invasion of PDAC cells *in vitro* **A.** Colo-357 and Capan-1 cells were treated with Scr and shMUC4, MUC4 protein levels were determined by Western blot analysis. **B.** MUC4 mRNA levels were detected by real-time PCR analysis. **C.** The migratory capabilities were evaluated by wound healing assays. Representative images of wound healing assays were shown. Original magnification, 40×; scale bar = 200 μm. **D.** The average area covered by migrating cells is presented. **E.** The migratory capabilities were determined by Transwell migration assays. Representative images are shown. Original magnification, 100×; scale bar = 200 μm. **F.** The number of migrated cells was quantified. **G.** The invasive capabilities were investigated by Transwell invasion assays. Representative images are shown. Original magnification, 100×; scale bar = 200 μm. **H.** The number of invasive cells was quantified. All data are presented as mean ± SEM. *p < 0.05, **p < 0.01, ***p < 0.001.

To evaluate the function of MUC4 in PDAC, we chose two cell lines with high MUC4 protein levels, Colo-357 and Capan-1, to generate cell lines with stable knockdown of MUC4 (Colo-357 shMUC4 and Capan-1 shMUC4). The MUC4 protein and mRNA levels significantly decreased in Colo-357 shMUC4 and Capan-1 shMUC4 cells (Figure 2A and 2B). Wound healing assay showed that MUC4 knockdown weakened the migration potential of Colo-357 and Capan-1 cells (Figure 2C and 2D). Next, we tested the migration and invasion of Colo-357 and Capan-1 cells by Transwell assays. Our data showed that the number of migrated cells decreased in Colo-357 shMUC4 and Capan-1 shMUC4 cells compared with the scramble shRNA (Scr)-treated cells (Colo-357 Scr and Capan-1 Scr) (Figure 2E and 2F). Furthermore, the number of invasive cells obviously decreased in Colo-357 shMUC4 and Capan-1 shMUC4 cells compared with the Scr-treated cells (Figure 2G and 2H). Taken together, these results suggested that MUC4 knockdown inhibits the migration and invasion of PDAC cell lines *in vitro*.

### MUC4 knockdown suppresses the migratory ability along the nerve of PDAC cells in a DRG-PDAC cell co-culture assay

**Figure 3:**
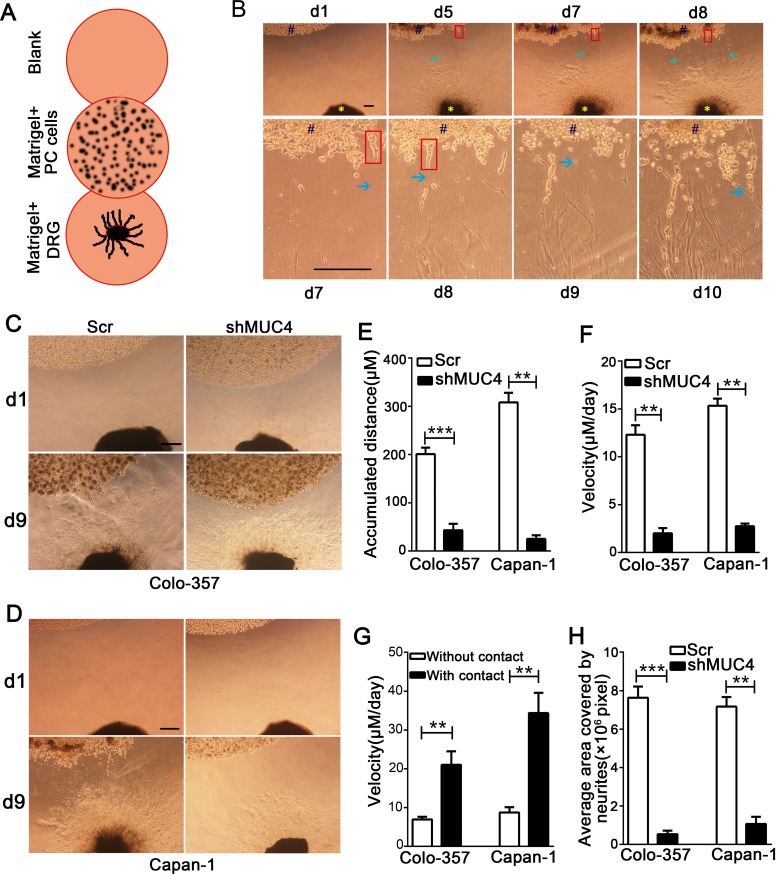
MUC4 knockdown suppresses the migratory ability along the nerve of PDAC cells in a DRG-tumor cell co-culture assay **A.** Schematic view of the DRG-tumor cell co-culture assay. PDAC cells were suspended in Matrigel and placed next to a DRG suspension. To exclude the possibility of non-specific PC cell migration, an additional Matrigel drop containing no neural cells (blank) was placed next to the other side of the PDAC cell suspension. **B.** Representative photomicrographs showing the entire process of the DRG-tumor cell interaction. The upper panel shows tumor cells that migrated towards the DRGs and neurites that projected towards the cancercell colonies (original magnification, 40×; scale bar=100 μm). The lower panel shows tumor cells that migrated along the neurites and neurites that projected into the cancer cell colonies (original magnification, 100×; scale bar = 100 μm). Blue # indicates cancer cell colony, yellow * indicates DRG, red rectangle shows tumor cell spikes, and green arrow points to a neurite. **C.** Representative day 1 (d1) and day 9 (d9) images of co-cultured with Colo-357 Scr and Colo-357 shMUC4 cells in the DRG-tumor cell co-culture assay. Original magnification, 40×; scale bar = 200 μm. **D.** Representative day 1 (d1) and day 9 (d9) images of co-cultured with Capan-1 Scr and Capan-1 shMUC4 cells in the DRG-tumor cell co-culture assay. Original magnification, 40×; scale bar = 200 μm. **E.** The accumulated distance travelled by the cancer cells was calculated. **F.** The travelling velocity of cancer cells was calculated. **G.** The difference between the travelling velocities of cancer cells with neurite and without neurite contact was analysed. **H.** The average area covered by the neurites growing out from the DRG was quantified. All data are presented as mean ± SEM. **p < 0.01, ***p < 0.001.

To investigate the interaction between PDAC cells and nerves, a modified co-culture assay of DRG and PDAC cells was established (Figure 3A). After DRGs and PDAC cells were co-cultured for 5 days, the tumor cells formed spikes heading the DRGs and started to detach from the cancer cell colonies. Concurrently, the axons significantly grew out from the DRGs and projected towards thecancer cell colonies (Figure 3B). After the cells were co-cultured for approximately 7 days, the tumor cells dissociated from the colony, came into contact with the axons and started to migratealong the neurites (Figure 3B). On the 9^th^day after starting the co-culture, we evaluated the accumulated travel distance and the migrating velocities of the cancer cells. The results showed that Colo-357 Scr and Capan-1 Scr cells travelled a greater distance compared with Colo-357 shMUC4 and Capan-1 shMUC4 cells (Figure 3C-3E). Moreover, Colo-357 Scr and Capan-1 Scr cells reached a higher migratingvelocity than Colo-357 shMUC4 and Capan-1 shMUC4 cells (Figure 3F). Furthermore, we found that the contact with neurites significantly sped up migrating velocity of cancer cells (Figure 3G).

Next, we analysed the neurites that grew out from the DRGs. We found that the average area covered by the neurites that co-cultured with Colo-357 shMUC4 and Capan-1 shMUC4 cells was smaller compared with that co-cultured with Scr-treated cells (Figure 3H). These results indicated that MUC4 knockdown in PDAC cells suppressed the outgrowth of neurites from the DRGs. This phenomenon also suggested that soluble nerve-derived factor(s) secreted by the cancer cells could be involved in favouring the interaction between cancer cells and nerves. Taken together, these data indicated that MUC4 knockdown inhibits the migratory potential of PDAC cells along the nerve.

### MUC4 knockdown weakens the NI of PDAC cells in vivo

**Figure 4:**
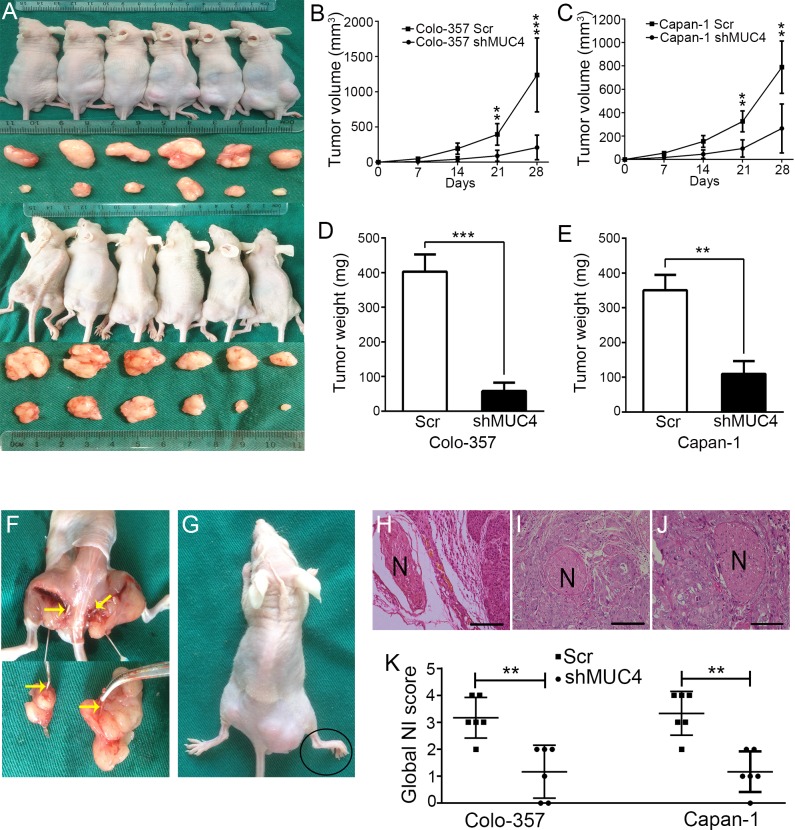
MUC4 knockdown suppresses NI by PDAC cells *in vivo* **A.** The NI model in nude mice. **B.** Tumor volumes in the Colo-357 groups on the indicated days. **C.** Tumor volumes in the Capan-1 groups on the indicated days. **D.** Tumor weights in the Colo-357 groups after excisions. **E.** Tumor weights in the Capan-1 groups after excisions. **F.** Anatomical images showing the sciatic nerves were encased in the xenografts. Yellow arrow points to the sciatic nerve. **G.** Representative images of hindlimb paralysis. Black oval shows the widthof paralyzed hind paw narrowed. (H, I, J) Images of three stages in themouse NI model. Original magnification, 200×; scale bar = 100 μm. **H.** NNI. **I.** EDA. **J.** PNI. ENI was not observed. **K.** Scatter plot showing the global NI scores. Differences were analysed by the Mann-Whitney *U*-test. **p < 0.01, ***p < 0.01.

A murine NI model was established by implanting cancer cells in the periphery of the sciatic nerve, which was used to assess the effect of MUC4 on NI *in vivo*. Four weeks after implantation, the xenografts were found to encase the sciatic nerve (Figure 4F). Tumor growth curves showed that the tumor volumes in the shMUC4-treatedgroup were significantly smaller compared with the Scr-treated group (Figure 4A-4C). Further analysis revealed that the tumor weights of the shMUC4-treated group were lighter than those of the control group (Figure 4D and 4E). These data confirmed our previously reported results [16].

To assess the effect of cancer cell invasion on sciatic nerve, we measured the hindlimb function and observed the nerve in tumor tissue slices under microscope. Modified NI scoring based on a previously described method was performed to estimate the severity of NI[18]. The modified NI score covered 2 parameters: (a) hindlimb behaviour and (b) micrography. For the hindlimb behaviour, a score of 0 was assigned for normal behaviour, 1 for gross behavioural signs of motor weakness and 2 for paralysis (Figure 4G). For micrography, a score of 0 was assigned when no neural invasion (NNI) (Figure 4H) was present, 1 was assigned for epineural tumor associations (ENAs) (Figure 4I), 2 for perineural invasion (PNI) (Figure 4J)and 3 for endoneural invasion (ENI) (not observed). The global NI scorewas obtained by adding the scores noted for each parameters (i.e., a + b). Our results showed that the global NI score of the MUC4 knockdown group was lower than that of the control group. That is to say, MUC4 knockdown weakens PDAC cell NI *in vivo* (Figure 4K).

### MUC4 mediates NI by regulating netrin-1 expression

**Figure 5:**
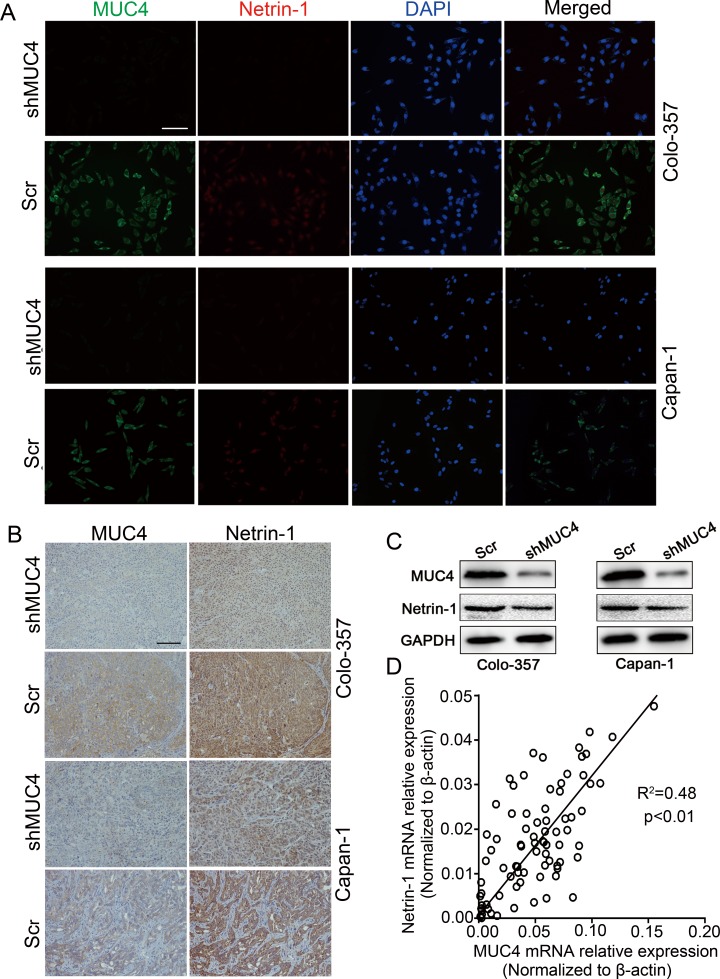
MUC4 regulates the expression of netrin-1 in PDAC **A.** Colo-357 and Capan-1 cells were treated with Scr and shMUC4, MUC4 and netrin-1 expressions were determined by immunofluorescence analysis. Alexa Fluor 488 (green) for MUC4, Alexa Fluor 594 (red) for netrin-1 andDAPI (blue) for nuclear staining. Original magnification, 200×; scale bar = 100 μm. **B.** MUC4 and netrin-1 expressions in nude mice xenografts were detected by immunohistochemicalanalysis. Original magnification, 200×; scale bar = 100 μm. **C.** Western blot analysis of MUC4 and netrin-1 expressions in Scr-treated and shMUC4-treated cells. **D.** Linear regression analysis was used to determine the correlation between the MUC4 and netrin-1 mRNA expression levels in human PDAC tissues. R^2^ = 0.48, *p* < 0.01, *n* = 82.

**Figure 6:**
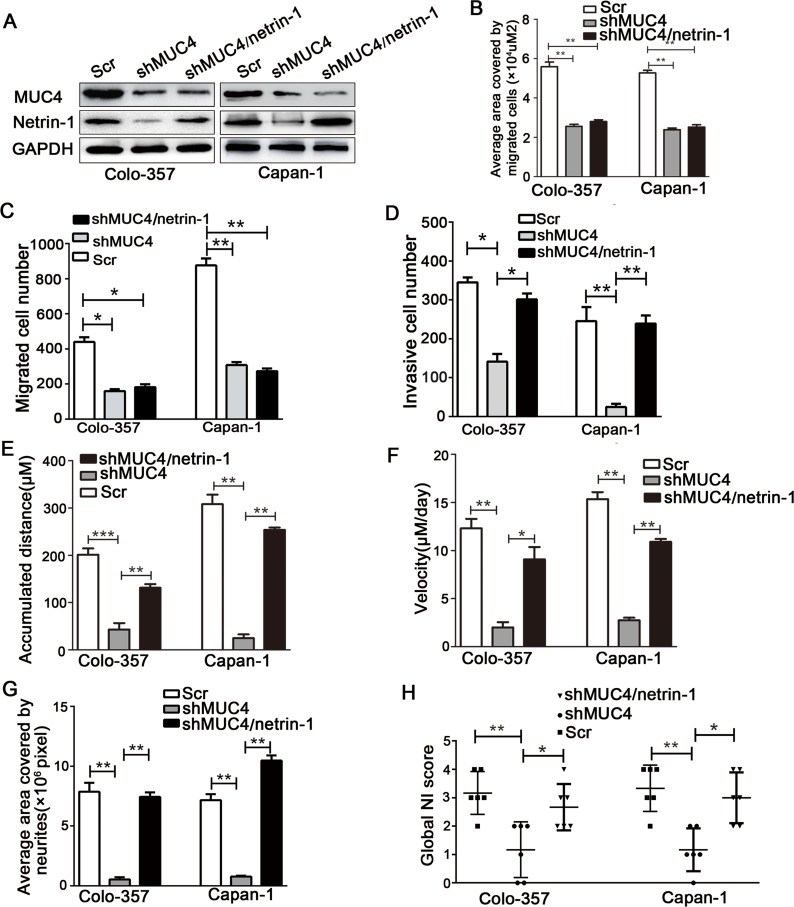
Exogenous overexpression of netrin-1 in shMUC4-treated cells restores their NI capability **A.** Western blot analysis of MUC4 and netrin-1 expressions in Colo-357 Scr, Colo-357 shMUC4, Colo-357 shMUC4/netrin-1, Capan-1 Scr, Capan-1 shMUC4 and Capan-1 shMUC4/netrin-1 cells. (B, C)The migratory capabilities wereevaluated by wound healing assays and Transwell migration assays, **B.** the average area covered by migrating cells is presented, **C.** the number of migrated cells was quantified. **D.** The invasive capabilities were investigated by Transwell invasion assays. The number of invasive cells was quantified. (E-G) The interaction between tumor cells and neurites was evaluated by the DRG-PDAC cell co-culture assay, **E.** the accumulated distance travelled by the cancer cells was calculated, **F.** the travelling velocity of cancer cells was calculated, **G.** the average area covered by the neurites growing out from the DRG was quantified. **H.** The NI capabilities in the mouse NI model were showed by Scatter plot. Differences were analysed by the Mann-Whitney *U*-test. The pooled data are presented as mean ± SEM. *p < 0.05, **p < 0.01.

To characterize the factors involved in the interaction between PDAC cells and nerves, we determined the differential expression levels of nerve-derived molecules such as GDNF (glial cell line-derived neurotrophic factor), NGF (nerve growth factor), CX3CL1 (chemokine ligand 1), netrin-1, artemin, and Slit-2 in shMUC4 and Scr treated cells by real-time PCR. This analysis revealed anapproximately twenty percent decrease in netrin-1 expression compared with the control cells (Supplementary Figure S2). The differential expression of netrin-1 in our experimental cancer cells was confirmed byimmunofluorescence and western blot analysis (Figure 5A and Figure 5C). We also assessed MUC4 and netrin-1 expression in tumor tissues from nude mice. These results showed that netrin-1 expression significantly decreased in Colo-357 shMUC4 and Capan-1 shMUC4 groups compared to theirrespective control groups (Figure 5B). Moreover, we analysed MUC4 and netrin-1 mRNA levels in human PDAC tissues. Linear regression analysis revealed that the relative expression level of netrin-1 correlated with that of MUC4 (Figure 5D). Therefore, we speculated that MUC4 promoted NI by regulating netrin-1 expression. To confirm our hypothesis, we performed rescue experiments by overexpressing netrin-1 in shMUC4-treated cells, to generate Colo-357shMUC4/netrin-1 and Capan-1 shMUC4/netrin-1 cells. The expression levels of netrin-1 were confirmed by western blot analysis (Figure 6A). Next, we evaluated the role of netrin-1 in NI *in vitro* and *in vivo*. Our results showed that the exogenous overexpression of netrin-1 restored all the abilities of shMUC4-treated cells to invade nerves except migratory capabilities (Figure 6B-6H). Taken together, these results demonstrated that MUC4 knockdown decreases netrin-1 expression in PDAC cells, leading to the inhibition of their NI potential.

### MUC4 regulates netrin-1 expression through the HER2/AKT/NF-κB pathway

**Figure 7:**
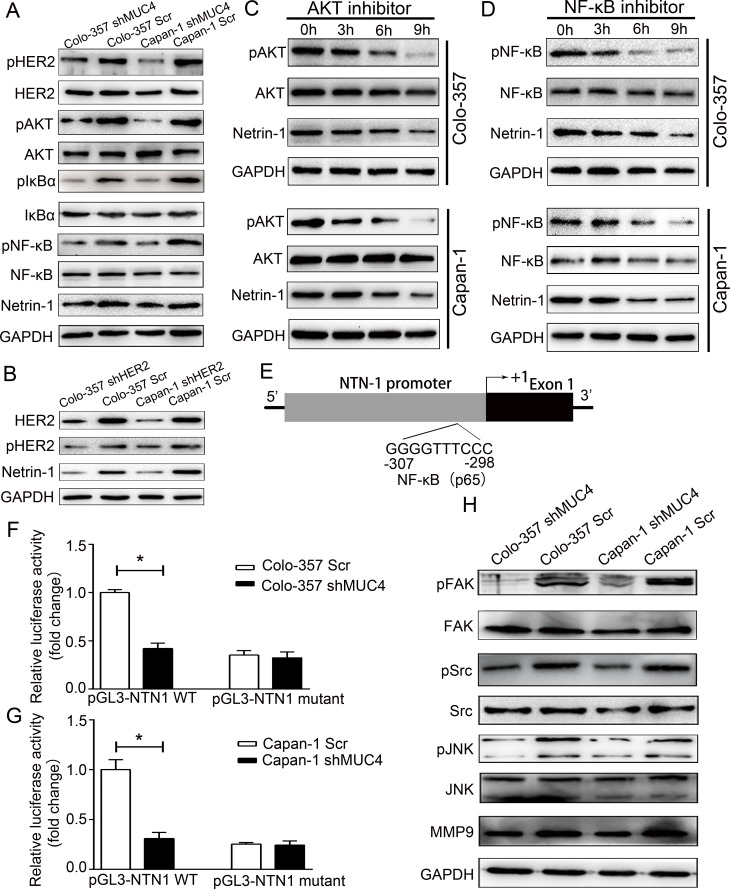
The downstream signaling pathways of MUC4 involved in the regulation of netrin-1 and in mediating the effect of shMUC4 on motility, migration and invasion **A.** Western blot analysis for pHER2, HER2, pAKT, AKT, pIκBα, IκBα, pNF-κB, NF-κB and netrin-1 in Scr-treated and shMUC4-treated cells, GAPDH was used as a control. **B.** Western blot analysis for pHER2, HER2 and netrin-1 in Scr-treated and shHER2-treated cells, GAPDH was used as a control. **C.** Colo-357and Capan-1 cells were treatment with AKT inhibitor for 0h, 3h, 6h and 9h. AKT, pAKT and netrin-1 protein levels were determined by western blot analysis. GAPDH was used as a control. **D.** Colo-357 and Capan-1 cells were treatment with NF-κB inhibitor for 0h, 3h, 6h and 9h. NF-κB, pNF-κB and netrin-1 protein levels were determinedby western blot analysis. GAPDH was used as a control. **E.** Schematic representation of the 5′-flanking NTN1 genomic region. Clonedpromoter (shaded box), first exons (black box), NF-κB (p65) binding site, which was identified using the TFSEARCH program, corresponding to nucleotides −307 to −298 (relative to the putative transcription start site +1) **F.** Scr-treated Colo-357 and shMUC4-treated Colo-357 cells were transfected with pGL3-NTN1 WT or pGL3-NTN1 mutant (preventing NF-κB binding) for 24 h and lysed for the luciferase activity assay. Promoter activity was normalized to pGL3-NTN1WT-transfected Colo-357 Scr cells. The data are presented as mean ± SEM. *p < 0.05. **G.** Scr-treated Capan-1 and shMUC4-treated Capan-1 cells were transfected with pGL3-NTN1 WT or pGL3-NTN1 mutant (preventing NF-κB binding) for 24 hand lysed for the luciferase activity assay. Promoter activity was normalized to pGL3-NTN1 WT-transfected Capan-1 Scr cells. The data are presented as mean ± SEM. *p < 0.05. **H.** Western blot analysis for pFAK, FAK, pSrc, Src, pJNK, JNK and MMP9 in Scr-treated and shMUC4-treated cells, GAPDH was used as a control.

To determine the mechanisms underlying the regulation of netrin-1 expression by MUC4 in Colo-357 and Capan-1 cells, we analysed the potential downstream signaling pathway affected by MUC4knockdown. Western blot analysis showed that the levels of HER2 activation (pHER2 levels) in Colo-357 shMUC4 and Capan-1 shMUC4 cells were significantly reduced, while the total HER2 levels remained unchanged (Figure 7A). To confirm HER2 was involved inregulation of the netrin-1 expression, we determined the netrin-1 expression in stable HER2 knockdown cell lines (Colo-357 shHER2 and Capan-1 shHER2). Compared to Colo-357 Scr and Capan-1 Scr cells, Colo-357 shHER2 and Capan-1 shHER2 cells reduced the expression of netrin-1 (Figure 7B). Our data also showed that AKT phosphorylation levels (pAKT) were lower in Colo-357 shMUC4 and Capan-1 shMUC4 cells compared to Colo-357 Scr and Capan-1 Scr cells, whereas thetotal AKT levels remained unchanged (Figure 7A). Then, we assessed the role of AKT in the regulation of netrin-1 in PDAC cells by treating Colo-357 and Capan-1 cells with an AKT inhibitor (10μmol/L, SC394003, Santa Cruz Biotechnology). Western blot analysis showed that treatment with an AKT inhibitor resulted in a time-dependentdecrease in pAKT and netrin-1 expression in Colo-357 and Capan-1 cells (Figure 7C). This finding confirmed the involvement of AKT in the MUC4-mediated regulation of netrin-1 expression. To determine whether NF-κB was involved in the regulation of netrin-1 by MUC4, we investigated the levels of pIκBα, total IκBα, pNF-κB and total NF-κB in Colo-357 shMUC4 and Capan-1 shMUC4 cells. We found that MUC4 knockdown resulted in decreased IκBα and NF-κB phosphorylation levels (Figure 7A). A time-dependent decrease in pNF-κB and netrin-1 expression levels in Colo-357 and Capan-1 cells treated with NF-κB inhibitor (5μmol/L, BAY 11-7082, Selleck, Shanghai, China) confirmed the role of NF-κB in the regulation of netrin-1 expression (Figure 7D). Furthermore, analysis of the NTN1 gene (encoding netrin-1) promoter using the TFSEARCH program revealed the presence of a NF-κB binding site(Figure 7E). To determine whether NF-κB directly affected the activity of the NTN1 promoter, Colo-357 shMUC4 and Capan-1 shMUC4 cells, along with their respective controls, were transfected with luciferase constructs containing either the wild-type NTN1 promoterwith an intact NF-κB binding site (pGL3-NTN1 WT) or a mutated NTN1 promoter with an inactivating mutation in the NF-κB binding site (pGL3-NTN1 mutant). Our data revealed that Colo-357 shMUC4 and Capan-1 shMUC4 cells transfected with the pGL3-NTN1 WT plasmid showed lower activities than the corresponding Scr-treated cells, whereas this difference was not observed when cancer cells were transfected with pGL3-NTN1 mutant construct (Figure 7F and 7G). Collectively, these results showed that MUC4 regulates netrin-1 expression via the HER2/AKT/NF-κB pathway.

### MUC4 knockdown reduces motility and invasion potentials through FAK, JNK and MMP9

We found that MUC4 knockdown reduced the motility and invasion potentials of PDAC cells. Thus, we were interestedin investigating the molecules or pathways associated with this phenomenon. Previous studies have demonstrated important roles for the focal adhesion kinase (FAK) and c-jun N-terminal kinase (JNK) pathways in cell motility [19]. Phosphorylated Src is the key mediator downstream of MUC4-mediated FAK activation [20]. Therefore, we examined FAK and Src expression in Colo-357 shMUC4 and Capan-1 shMUC4 cells and in Scr-treated Capan-1 and Colo-357 cells by western blot analysis. These results revealed that pFAK and pSrc expression was significantly decreased in both Colo-357 shMUC4 and Capan-1 shMUC4 cells when compared with their respective control cells, while no change was observed in their total protein levels (Figure 7H). This observation was similar to the results of previous studies [21, 22]. Then, we analysed the protein expression levels of JNK and pJNK and found that MUC4 knockdown leads to a decrease in pJNK levels in both Colo-357 shMUC4 and Capan-1 shMUC4 cells. However, no change was observed in their total protein levels (Figure 7H). Furthermore, we observed that the expression levels of matrix metalloproteinase-9 (MMP9), a key molecule associated with the invasion potential of tumor cells, were decreased in Colo-357 shMUC4 and Capan-1 shMUC4 cells (Figure 7H).

## DISCUSSION

NI is a special pattern of invasion and a characteristic pathological feature of PDAC, which correlates with the progression and prognosis in PDAC [23]. Different from conventional invasion, NI of PDAC includes two special aspects, i.e., neurotropism of pancreatic cancer cells and motivation ofnerve-derived molecules in the interaction between nerve and PDAC cells[24]. Previous studies have shown that MUC4 enhances PC cell proliferation, apoptosis, motility, invasion, metastasis and drug resistance [20, 25-27]. However, the underlying molecular mechanisms and functional role of MUC4 in NI of PDAC remains unclear. In this study, we firstly found a positive correlation between MUC4 expression levels and NI in human PDACtissues. Furthermore, we showed for the first time that MUC4 knockdown inhibited the NI of PDAC cells *in vitro* and *in vivo*. In addition, we identified that nerve-derived molecule netrin-1 mediates the NI reinforced by MUC4 via HER2/AKT/NF-κB pathway in PDAC. To our knowledge, this is the first report that netrin-1 involved in NI of PDAC.

Previous studies showed that the transmembrane mucin MUC4 is aberrantly overexpressed in PDAC [28, 29]. In correspondence with the studies, we found that MUC4 expression levels in PDAC tissues were significantly higher than those in the adjacent tissues. Aloysius et al showed that high expression of MUC4 wasassociated with a higher postoperative recurrence rate than low expression and with reduced survival in PC [30]. Our data indicated that MUC4 expression significantly correlated with the T grade and tumor stage. Furthermore, we identified that the human PDAC tissues presence of NI exhibited a higher MUC4 expression level compared to non-neurally invasive PDAC tissues. Conversely, high MUC4 expression of in tumor tissues indicated a higher incidence of NI than low MUC4 expression. Taken together, our results demonstrated that MUC4 expression level positively correlates with NI, suggesting MUC4 plays a functional role in NI in PDAC.

To uncover the functional role of MUC4 in PDAC NI, we selectively silenced MUC4 expression in Colo-357 and Capan-1 cells, which express higher levels of MUC4 than other pancreatic cancer cell lines. Then, we investigated the effect of MUC4 knockdown on NI using various functional assays *in vitro* and *in vivo*. The NI potential of cancer cells is associated with their motility, migration, invasion and neurotropic potential [24]. Our data showed that MUC4 knockdown inhibited cell motility, migration and invasion as determined by wound healing and Transwell migration assays. Furthermore, we also observed the downregulation of pFAK, pSrc, pJNK and MMP9 in Colo-357 shMUC4 and Capan-1 shMUC4 cells compared to control cells. These findings confirmed previous studies reporting that MUC4 promoted the motility and invasive potentials of cancer cells.

To investigate the mutual tropism between neurons and PDAC cells in the NI process, we performed the DRG-tumor cell co-culture assay, which has emerged as a promising model to identify NI mediators [24]. We found that MUC4 knockdown decreased the accumulated migration distance and the velocity of cancer cells. In addition, we also found that MUC4 knockdown inhibited DRG neurite outgrowth. This finding is in accordance with a previous study reporting that PC cell proliferation and growth positively correlated with the extent of DRG neurite outgrowth [31]. This result suggested that MUC4 could reinforce the neurotrophic effectof pancreatic cancer cells towards neurons. Because of the membrane localization of MUC4, we speculated that a nerve-derived factor might mediate the effect of MUC4 on NI. In addition, our data showed that oncein contact with neurites, the cancer cells migrated more quickly along neurites than cells without neurite contact. This finding suggested thatNI promoted cancer progression. To evaluate the interaction between PDAC cells and neurons *in vivo*, we useda murine model of NI, which further emphasized the capacity of MUC4 to enhance NI. Overall, we found that MUC4 knockdown suppresses the neural invasive potential of PDAC cells.

Netrin-1 is a secreted protein that regulates axon guidance and neuronal migration [32, 33]. In non-neural tissues, netrin-1 has a variety of functions, including promoting cell adhesion, invasion, metastasis, angiogenesis and survival[34-37]. Dumartin and colleagues showed that netrin-1 increases the invasive potential and confers apoptosis resistance in PC [38]. To assess whether netrin-1 mediated the effect of MUC4 on PDAC NI, we investigated netrin-1 expression in Colo-357 shMUC4 and Capan-1 shMUC4 cells. Our data showed that netrin-1 expression paralleled MUC4 expression in experimental cells as determined by immunofluorescence double staining assay. We obtained a similar result via immunohistochemical analysis of xenografts obtained from the murine NI model. Furthermore, by analysing mRNA expression in human PDAC tissues, we found that MUC4 expression correlated with netrin-1 expression. Thesefindings suggested that netrin-1 is a downstream target of MUC4. To further confirm whether netrin-1 mediates the effect of MUC4 on PDAC NI, we evaluated the effect of re-expressing netrin-1 in shMUC4-treated cells. We found that netrin-1 re-expression restored the NI potential ofColo-357 shMUC4 and Capan-1 shMUC4 cells. Taken together, our results suggested that the axon guidance factor netrin-1 mediates the MUC4-induced effects on PDAC NI.

Previous studies have shown that MUC4 is an intramembrane ligand activator of HER2 that facilitates the dimerizationof HER2 with other ErbB receptors [21, 39]. In addition, MUC4 was also shown to interact with HER2 and to activate the PI3K/AKT/NF-κB pathway [40]. Paradisi revealed that NF-κB regulates netrin-1 expression in human colorectal cancer cells [41]. Based on these studies, we speculated that the HER2/AKT/NF-κB pathway could participate in the regulation of netrin-1 in pancreatic cancer. Weobserved HER2 downregulation in shMUC4-treated cells and the reduced expression of netrin-1 in shHER2-treated cells. The result indicated HER2 was involved in the regulation of netrin-1 expression. Besides, theresult also supported trastuzumab, a HER2-targeted agent, as a plausible drug for treating NI of PDAC. Then, we investigated the effectof MUC4 knockdown on AKT and NF-κB phosphorylation. The canonical NF-κBpathway is activated by IκBα phosphorylation, resulting in the liberation and phosphorylation of NF-κB [42]. We observed pIκBα and pNF-κB downregulation in shMUC4-treated cells. A time-dependent decrease in netrin-1 expression was induced by AKT and NF-κB inhibitors, confirming that AKT and NF-κB are key mediators of netrin-1 regulation. Furthermore, our analysis of the netrin-1 promoter revealed the presence of a NF-κB binding site. Then, we confirmed the direct effect of NF-κB on the NTN1 promoter in PDAC cells by luciferase reporter assay. Taking these results together, we concluded that MUC4 modulated the expression of netrin-1 via the HER2/AKT/NF-κB pathway.

**Figure 8:**
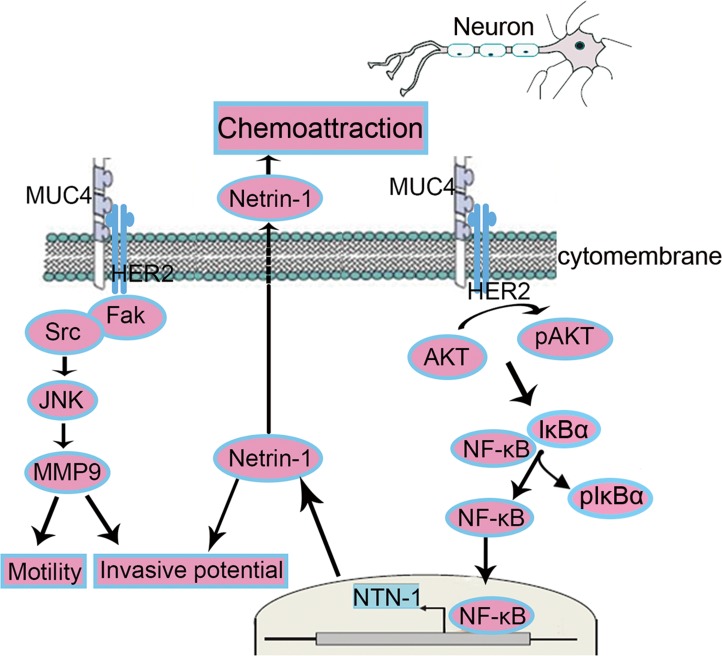
Schematic representation of the proposed mechanisms of MUC4-modulated NI Netrin-1-independentmechanism: MUC4 promotes the motility and invasion potentials of PDAC cells via the FAK, JNK and MMP9. Netrin-1-dependent mechanism: MUC4 increases the expression of netrin-1 via the HER2/AKT/NF-κB pathway in PDAC cells, enhancing the potential of NI and chemoattraction.

Globally, our studies provided evidence that MUC4 regulates netrin-1 expression via the HER2/AKT/NF-κB pathway, leading to an increased neural invasive potential (Figure 8). Recent studies have showed that miR-219-1-3p and targeting epidermal growth factor receptor (EGFR) - signal transduction and activation of transcription-1 (STAT1) axis decreased the expression of MUC4 in PC [43, 44]. Heather et al. showed O-glycosylation domain of MUC4 participated in its antiapoptotic activity [26]. Further analyses focusing on regulating the expression and posttranscriptional modification of MUC4 are promising for better understanding and treatment of PDAC-associated NI.

## MATERIALS AND METHODS

### Samples and patients

Pancreatic tissue samples were collected from 82patients who underwent radical resection for PDAC at the First Affiliated Hospital of Nanjing Medical University or at the Taizhou People's Hospital, China, between 2008 and 2013. No chemotherapy or radiation therapy was administered before surgery. Written informed consent was obtained from all patients or from their relatives. This study was approved by the Ethics Committees of Nanjing Medical University.

### Cell lines and cell culture

The human PDAC cell lines (Mia PaCa, CFPAC, BxPC-3, Capan-1, Colo-357, and HPAC) were purchased from the Shanghai Cell Bank (Shanghai, China). These cells were grown in Dulbecco's modified Eagle's medium (DMEM) (Gibco, Grand Island, NY, USA) supplemented with 10% fetal bovine serum (FBS) (Gibco), 100 mg/ml penicillin and 100 mg/ml streptomycin (Gibco) at 37°C with 5% CO_2_.

### RT-PCR and real-time PCR

Total RNA from pancreatic cancer cells was extracted using TRIzol reagent (Invitrogen, Carlsbad, CA, USA), and cDNAwas synthesized using PrimeScript RT Reagent (Takara, Dalian, China) according to the manufacturer's protocol. Gene expression levels were normalized to the expression level of β-actin. Each PCR was performed intriplicate and independently repeated three times. The primer sequences are showed in Supplementary Table 1.

### RNA interference and plasmids

ShRNA targeting MUC4 (5′-AACGCAAGCATCGGACTTCAC-3′), shRNA targeting HER2 (5′-GAGTCCCAACCATGTCAAA-3′) and the scramble shRNA (5′-GTTCTCCGAACGTGTCACGT-3′) were synthesized and ligated into the retroviral pGPU6/GFP/Neo vector (GenePharma, Shanghai, China). Retrovirus packaging and transfection were conducted according to the manufacturer's instructions. Full-length netrin-1 cDNA was synthesized and cloned into the pGCMV/MCS/Neo vector (GenePharma). *E. coli* TOP10 (GenePharma) competent cells were used for plasmid amplification. Plasmid DNA was transfected into cancer cells using Lipofectamine 2000 reagent (Invitrogen).

### Immunohistochemical analysis

Immunohistochemistry was performed as described previously [16]. The following primary antibodies were used: MUC4 8G7, PGP9.5 (Abcam, Cambridge, UK; 1:400 dilution), and netrin-1 (Santa Cruz Biotechnology, CA, USA; 1:200 dilution). Expression levels were evaluated according to the staining intensity (0 for absent, 1 for weak, 2 for moderate and 3 for strong staining). The proportion of positive cells (0 for < 10%, 1for 10 to < 50%, and 2 for ≥ 50% of cells) was also assessed. The sum of the intensity and proportion scores was used to analyse the immunoreactivity levels.

### Western blot analysis

Pancreatic cancer cells were processed for protein extraction according to standard procedures. Extracted proteins were resolved by electrophoresis. For MUC4, the proteins were performed on SDS-agarose (2%) gel under reducing conditions. For netrin-1, GAPDH, Src, HER2, MMP9 FAK, AKT, JNK, NF-κB (p65) and IκBα, SDS-polyacrylamide gel electrophoresis (8-10%) was performed under similar conditions. Then, the resolved proteins were transferred to polyvinylidene difluoride membranes (PVDF). Non-specific protein interactions were blocked by incubation in 5% non-fat dry milk in TBST buffer [20 mM Tris-HCl, 150 mM NaCl, 0.1% Tween 20 (pH 7.6)] at room temperature for 2h. Then, the membranes were incubated with primary antibodies at 4°C overnight. After the membranes were washed with TBST, they were incubated with horseradish peroxidase-conjugated secondary antibodies. Proteins were visualized using a SuperSignal West Femto Maximum Sensitivity Substrate Kit (Thermo Scientific, Logan, UT). Western blots were quantified using the Image-Pro Plus software version 6. The following primary antibodies were used: MUC4 1G8, anti-MMP9, netrin-1 (Santa Cruz Biotechnology; 1:200 dilution), GAPDH, pSrc, Src, pHER2, HER2, pFAK, FAK, pAKT, AKT, pJNK, JNK, pNF-κB (p65), NF-κB (p65), pIκBα and pIκBα (Cell Signaling Technology, Danvers, MA; 1:1000 dilution).

### Wound healing assay

In total, 3 × 10^5^cells were seeded in 6-well Petri dishes and allowed to reach approximately 90% confluence. A scratch was made in each well using sterile 200-μl pipet tips. Subsequently, the cells were washed and incubated in growth medium for 24 h at 37°C with 5% CO_2_. The scratches were documented, and the migration rates were calculated at 24 h. The experiments were performed in triplicate; representative images are reported.

### Transwell assay

For invasion assays, the upper surface of each insert was coated with Matrigel (St Louis, MO, USA) according to the manufacturer's protocol. Complete culture medium containing 10% FBS was used as the chemoattractant in the lower chamber. In total, 5 × 10^4^ cells were seeded in the upper chamber in serum-free medium and incubated at 37°C under a 5% CO_2_-saturatedhumidified atmosphere. Cell migration was assessed at 24 h; invasion, at 48 h. The non-invading cells were gently wiped from the upper surfaceof the membrane with cotton-tipped swabs. Cells on the lower surface ofthe membrane were stained with 0.1% crystal violet for 20 min and counted. The experiments were performed in triplicate. The same experimental design was used for the migration assay, but the membranes were not pre-coated with Matrigel.

### DRG-tumor cell co-culture assay

DRG-tumor cell co-culture assay was performed using a modified method based on a previously described protocol [45]. In total, 2 × 10^5^PDAC cells were suspended in 25 μL of growth-factor-reduced Matrigel (Sigma-Aldrich) and placed at the centre of each well of 12-well Petri dishes. After anterior laminectomy, DRGs were isolated from the lumbar spinal region of 8-day-old Sprague-Dawley rats and then seeded in 25 μL of Matrigel next to the cell suspension. To exclude the possibility of non-specifically guided migration of pancreatic cancer cells, an additional 25 μL of blank Matrigel was positioned on the opposite side. The Petri dishes were incubated for 30 minutes at 37°C in a humidified 5% CO_2_-saturated atmosphere to allow Matrigel polymerization. After solidification, neurobasal medium (Invitrogen) supplemented with 10% FBS, 100 U/mL penicillin, 100 μg/mL streptomycin, 0.5 mM L-glutamine and 2% B-27 (Invitrogen) was added to the wells and renewed every 2 days.

### *In vivo* NI model

All animal experiments were conducted according to the guidelines of the Nanjing Medical University Institutional AnimalCare and Use Committee. An *in vivo* NI model was generated using a modified method based on a previously described protocol [46]. Four-week-old male nude mice were anesthetized by methoxyflurane inhalation for the procedures. The sciatic nerve, which is deep under the femoro coccygeus and biceps femoris muscles, was surgically exposed. In total, 5 μL of PDAC cell suspension containing 2 × 10^5^/μLwere injected into the periphery of the sciatic nerve using a 100-μL syringe. For assessing the effect of MUC4 knockdown on NI in Colo-357 and Capan-1 cells, twelve mice (BALB/c-nu (nu/nu)) were purchased from the Shanghai Experimental Animal Centre (Chinese Academy of Sciences, Shanghai, China) and divided into two groups (six mice /group). ShMUC4-treated cells were injected into the left pygal; the Scr-treated cells, into the right pygal. Tumor volumes and sciatic nerve-related hindlimb behaviours were evaluated every 7 days. Mice were euthanized after 6 weeks, xenografts were excised. NI severity was assessed by hindlimb behaviours and histological analyses. The same experimental design was used to assess the effect of netrin-1 re-expression on NI in shMUC4-treated cells. ShMUC4-treated cells were injected into the left pygal; the shMUC4/netrin-1-treated cells, into the right pygal.

### Immunofluorescence double staining assay

To study the localization and effect of MUC4 knockdown on netrin-1 expression, immunofluorescence double staining assay was performed. Cells were grown on sterile coverslips in 12-well plates, fixed in 3.7% paraformaldehyde for 20 min at room temperature, and incubated with anti-MUC4 1G8 and anti-Netrin-1 (Santa Cruz Biotechnology; 1:50 dilution). The secondary antibodies used were Alexa Fluor 488 and Alexa Fluor 594 (Invitrogen; 1:1000 dilution). 4′-6-Diamidino-2-phenylindole (DAPI) was applied to the samples after the final wash to visualize the nuclei. Images were visualized using a Zeiss fluorescence microscope.

### Luciferase reporter assay

Luciferase reporter assays were performed using aDual-Luciferase Reporter Assay System (Promega, Madison, WI, USA). In total, 5 × 10^3^ cells/wellwere plated in 96-well plates. The cells were serum starved for 6 h andtransfected with 0.2 μg of a promoter reporter construct containing either an intact NF-κB binding site (pGL3-NTN1 WT) or a mutation that prevents NF-κB binding (pGL3-NTN1 mutant) (Millipore, Temecula, CA, USA). In total, 0.1 g of *Renilla* luciferase plasmid (Promega) was transfected as a control using Lipofectamine 2000 reagent. Firefly and *Renilla* luciferase activities were measured at 24 h after transfection according to the manufacturer's instructions. Each experiment was performed in triplicate.

### Statistical analysis

Statistical analysis was performed using SPSS (Statistical Package for the Social Sciences) software version 20.0. Unless otherwise indicated, the differences between groups were analysedusing Student's *t*-test. The data are presented as the mean ± SEM and considered significant when *p < 0.05, **p < 0.01 or ***p < 0.001.

## SUPPLEMENTARY MATERIAL FIGURES AND TABLE


