# Supplementary material for: MUC4-promoted neural invasion is mediated by the axon guidance factor netrin-1 in PDAC
Source: Oncotarget. 2015 Sep 15;6(32):33805–22. doi: 10.18632/oncotarget.5668 (PMC4741804; doi:10.18632/oncotarget.5668)
Supplement: Supplementary file 1 [file oncotarget-06-33805-s001.pdf]

MUC4-promoted neural invasion is mediated by the axon guidance factor netrin-1 in PDAC

Supplementary Material

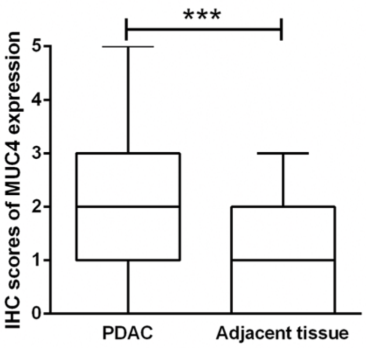

Supplementary Figure S1

Box plot showing the immunohistochemistry (IHC) scores for MUC4 protein expressions in 82 PDAC tissues and paired adjacent tissues. Differences were analysed using the Mann-Whitney *U*-test. *** indicates $p < 0.001$.

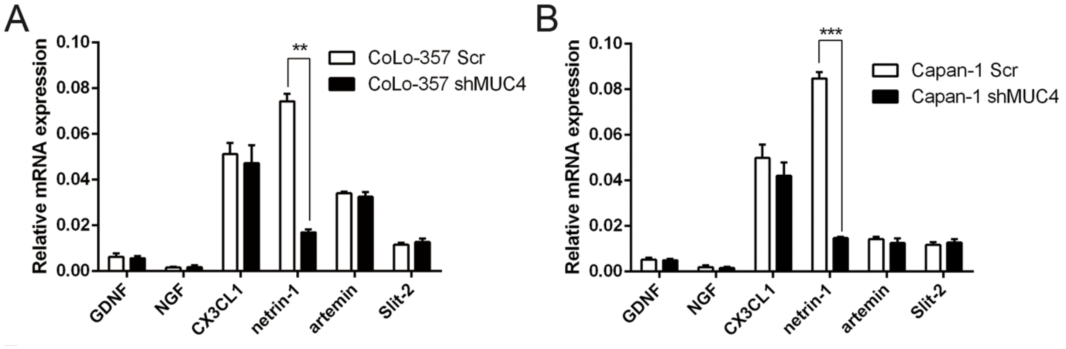

Supplementary Figure S2

(**A**) Real-time RT-PCR analyses of six nerve-derived molecules (GDNF, NGF, CX3CL1, netrin-1, artemin and Slit-2) mRNA levels in Colo-357 Scr and Colo-357 shMUC4 cells. (**B**) Real-time RT-PCR analyses of six nerve-derived molecules (GDNF, NGF, CX3CL1, netrin-1, artemin and Slit-2) mRNA levels in Capan-1 Scr and Capan-1 shMUC4 cells. The data are presented as mean ± SEM. ** indicates $p < 0.01$, *** indicates $p < 0.001$.

Supplementary Table 1: Primer sequences for amplification of target genes

Gene	Forward	Reverse
MUC4	GAGGAATGACCAGCTGCCTT	AGGGCCAGGGTGTCATAGAT
NTN1	GCATGCAGGTTGCAGTTACA	GCTGCAAGCCCTTCCACTA
GDNF	CCGACCTTTTCCTCTGGAAT	AAGTTATGGGATGTCGTGGC
NGF	AATCAACTCCTGCTTGGC	GTATTTAGCCCCTCCTCC
ARTN(encoded artemin)	CTCTATCAACACCAGGAGCG	CGGAAAGGTGCCTAGAAGAA
CX3CL1	ACGTGATGTTGCATTTCGTC	CCGATATCTCTGTCGTGGCT
Slit2	TTTTTATGTCAACTGCCCCAC	TCTTGGGACTGCGAAGCTAT
β-Actin	GACTTAGTTGCGTTACACCCTTTCT	GAACGGTGAAGGTGACAGCAGT
